# Investigation of Macrolide Resistance Genotypes in *Mycoplasma bovis* Isolates from Canadian Feedlot Cattle

**DOI:** 10.3390/pathogens9080622

**Published:** 2020-07-30

**Authors:** Andrea Kinnear, Tim A. McAllister, Rahat Zaheer, Matthew Waldner, Antonio C. Ruzzini, Sara Andrés-Lasheras, Sarah Parker, Janet E. Hill, Murray D. Jelinski

**Affiliations:** 1 Western College of Veterinary Medicine, University of Saskatchewan, Saskatoon, SK S7N 5B4, Canada; andrea.kinnear@usask.ca (A.K.); matthew.waldner@usask.ca (M.W.); antonio.ruzzini@usask.ca (A.C.R.); sarah.parker@usask.ca (S.P.); janet.hill@usask.ca (J.E.H.); 2 Lethbridge Research and Development Centre, Agriculture and Agri-Food Canada, Lethbridge, AB T1J 4B1, Canada; tim.mcallister@canada.ca (T.A.M.); rahat.zaheer@canada.ca (R.Z.); sara.andreslasheras@canada.ca (S.A.-L.); 3 College of Medicine, Department of Biochemistry, Microbiology and Immunology, University of Saskatchewan, Saskatoon, SK S7N 5E5, Canada

**Keywords:** antimicrobial, susceptibility, resistance, genotype, rRNA, macrolides, feedlot, beef, cattle

## Abstract

*Mycoplasma bovis* is associated with bovine respiratory disease (BRD) and chronic pneumonia and polyarthritis syndrome (CPPS) in feedlot cattle. No efficacious vaccines for *M. bovis* exist; hence, macrolides are commonly used to control mycoplasmosis. Whole genome sequences of 126 *M. bovis* isolates, derived from 96 feedlot cattle over 12 production years, were determined. Antimicrobial susceptibility testing (AST) of five macrolides (gamithromycin, tildipirosin, tilmicosin, tulathromycin, tylosin) was conducted using a microbroth dilution method. The AST phenotypes were compared to the genotypes generated for 23S rRNA and the L4 and L22 ribosomal proteins. Mutations in domains II (nucleotide 748; *E. coli* numbering) and V (nucleotide 2059 and 2060) of the 23S rRNA (*rrl*) gene alleles were associated with resistance. All isolates with a single mutation at Δ748 were susceptible to tulathromycin, but resistant to tilmicosin and tildipirosin. Isolates with mutations in both domain II and V (Δ748Δ2059 or Δ748Δ2060) were resistant to all five macrolides. However, >99% of isolates were resistant to tildipirosin and tilmicosin, regardless of the number and positions of the mutations. Isolates with a Δ748 mutation in the 23S rRNA gene and mutations in L4 and L22 were resistant to all macrolides except for tulathromycin.

## 1. Introduction

*Mycoplasma bovis* is associated with various diseases of cattle such as pneumonia, mastitis, arthritis, otitis media, conjunctivitis, and reproductive disorders [1,2]. In feedlot cattle, *M. bovis* infections commonly manifest as bovine respiratory disease (BRD) and chronic pneumonia and polyarthritis syndrome (CPPS) [3,4]. Furthermore, *M. bovis* infections often respond poorly to antimicrobial therapy, resulting in a chronic infection [5]. This lack of a response frequently results in prolonged antimicrobial therapy, which indiscriminately selects for antimicrobial resistance in the pathogens that comprise the BRD complex [6]. Mycoplasmosis in the feedlot results in economic losses due to reduced production performance, increased treatment costs, and death loss [2,6]. In addition, feedlot cattle with polyarthritis may become severely lame, which is a significant animal welfare issue.

As there are currently no effective vaccines for *M. bovis*, antimicrobials remain the primary means for the prevention and control of mycoplasmosis [2,7]. This has led to a number of *M. bovis* antimicrobial susceptibility studies in Canada [8,9,10,11], United States [12], Japan [13] and Europe [7,14,15,16,17,18,19]. These studies suggest that *M. bovis* will continue to become increasingly resistant to antimicrobials. This situation is exacerbated by the limited number of antimicrobials available for treating mycoplasma infections. *Mycoplasma* spp. lack a cell wall and the ability to synthesize folate, rendering them intrinsically resistant to all β-lactams and sulfonamides [2]. In addition, most aminoglycosides either lack label claims for BRD, or the formulations are not amenable for use in feedlot cattle. This narrows the selection of antimicrobials to those that target protein synthesis or DNA replication, and that have been formulated to maintain therapeutic blood levels for several days. The main class of antimicrobials that meet these criteria is the macrolides. 

Macrolides have been formulated to be administered parenterally or in-feed; however, only one macrolide, tylosin tartrate (TYLT), is registered in Canada for in-feed use. Tylosin is typically administered throughout the feeding period, and is used to control liver abscesses [9]. The other four main macrolides used in the feedlot are: tilmicosin (TIL), tildipirosin (TIP), tulathromycin (TUL), and gamithromycin (GAM). All of which are formulated as long-acting injectable antimicrobials, and depending on the drug, may have label claims for the control (metaphylaxis) and treatment of BRD. A distinctive pharmacological characteristic of macrolides that makes them ideally suited for use in feedlot cattle is their predilection to concentrate in the pulmonary epithelial fluid [20]. This is notable because BRD is the most prevalent and costly disease of feedlot cattle [21]. Thus, the macrolides’ pharmacokinetic and pharmacodynamic profiles are particularly well suited for metaphylaxis therapy for BRD in feedlots [22]. In western Canada, cattle deemed to be a high risk for developing BRD often receive TUL at the time of arrival to the feedlot; whereas, low risk cattle may receive either no antimicrobials or a long-acting oxytetracycline [23]. Lastly, unlike other BRD pathogens, antimicrobial resistance in *M. bovis* is not associated with antimicrobial resistance genes [24], but rather resistance arises from mutations in ribosomal RNAs [25].

Macrolides are a member of the macrolide–lincosamide–streptogramin B (MLS_B_) superfamily, all of which exert a bacteriostatic effect by disrupting protein synthesis [26]. Specifically, they bind with domains II and V of 23S rRNA, which is a component of 50S ribosomal subunit [27,28]. Ribosomal proteins L4 and L22 are positioned in close proximity to these macrolide binding sites [28,29]. Mutations within 23S rRNA and the L4 and L22 ribosomal proteins are associated with macrolide resistance [25,30]. This mechanism of resistance is not unique to *M. bovis* [13,31,32], having been reported in a variety of bacterial species, including other *Mycoplasma* spp. [33,34], *Neisseria gonorrhoeae* [30], *Streptococcus* spp. [35,36], *Francisella tularensis* [37], *Escherichia coli* [38], *Chlamydia trachomatis* [39], and *Haemophilus influenzae* [40].

A limitation of antimicrobial susceptibility testing (AST) for *M. bovis* is the lack of established clinical breakpoints from the Clinical and Laboratory Standards Institute (CLSI) and the European Committee on Antimicrobial Susceptibility Testing (EUCAST). As a result, researchers have extrapolated *M. bovis* clinical breakpoints from human *Mycoplasma* spp. and other bovine respiratory pathogens for which clinical breakpoints have been established [9,11,12,15,41,42,43]. Another challenge with performing AST on *M. bovis* is its very fastidious culture requirements, which is related to its reduced genome and limited biosynthetic capacity [44]. These requirements, coupled with relatively slow nonprolific growth, have encouraged the development of rapid molecular testing techniques for predicting antimicrobial susceptibility for *M. bovis* [13,45]. Utilization of a genotypic approach to assess antimicrobial susceptibility of *M. bovis* could allow for more expeditious evaluation of antimicrobial efficacy and evidence-based selection of antimicrobials to enable judicious use of antimicrobials, which are all principles of antimicrobial stewardship. Additionally, a genotypic approach could be more amenable as a standardized approach to assess antimicrobial susceptibility in *M. bovis* than culture-based techniques, as it would not be susceptible to variable results due to growth conditions. To support these efforts, this study assessed the concordance between genotypes known to confer macrolide resistance to AST phenotypes. Specifically, the study compared the 23S rRNA, L4, and L22 genotypes of *M. bovis* isolates to the AST results of five macrolides commonly used in western Canadian feedlot cattle to control and treat BRD.

## 2. Results

### 2.1. Culture and Reference Antimicrobial Susceptibilities

A total of 126 *Mycoplasma bovis* isolates were derived from 96 head of feedlot cattle from 21 feedlots over 12 production years, 2006 to 2018 (Table 1). Thirty head of cattle provided paired lung/joint isolates (*n* = 60), 14 provided a lung sample, 5 provided a joint isolate, and 47 isolates came from the nasopharynx. Nasopharyngeal isolates were derived from healthy (*n* = 30), diseased (*n* = 15), and dead (*n* = 2) cattle. Phenotypically resistant isolates to the macrolides tested were derived from samples taken from healthy, diseased, and dead cattle (Table 2). Production years were used to define the sampling cohort, as animals often enter the feedlot in the fall and remain until the following calendar year. Thus, the 2018 production year included samples obtained between 1 November 2018 and 30 June 2019.

*Mycoplasma bovis* PG45 (*Mycoplasma bovis* ATCC^®^ 25523) was resequenced and possessed the equivalent 23S rRNA genotype at positions 748, 2059, and 2060, as reported in the published reference genome [46]. Compared to the published reference genome, no nonsynonymous mutations in L4 and L22 ribosomal proteins were observed in this resequenced isolate. The following minimum inhibitory concentration (MIC) values, defined as the lowest concentration of antimicrobial to visibly inhibit growth, were determined from AST of five PG45 replicates: GAM, 8–16; TIP, 4–8; TIL, 1; TUL, 0.25; and TYLT, 1–2 µg/mL. Due to these genotypic and phenotypic findings, *M. bovis* PG45 was considered to be a susceptible wildtype isolate.

### 2.2. Genome Sequencing and Assembly

Draft genomes of the 126 isolates were assembled from an average 210,113 paired reads (range: 55,951 to 414,042); average read length of 217 bp (range: 166 to 233 bp). This produced assemblies with an average N50 of 18,690 bp (range: 1780 to 34,113 bp), an average coverage depth of 45.3 (range 12.2 to 89.1), and an average of 579 contigs (range: 171 to 1639).

### 2.3. 23 S rRNA Gene

**Figure 1:**
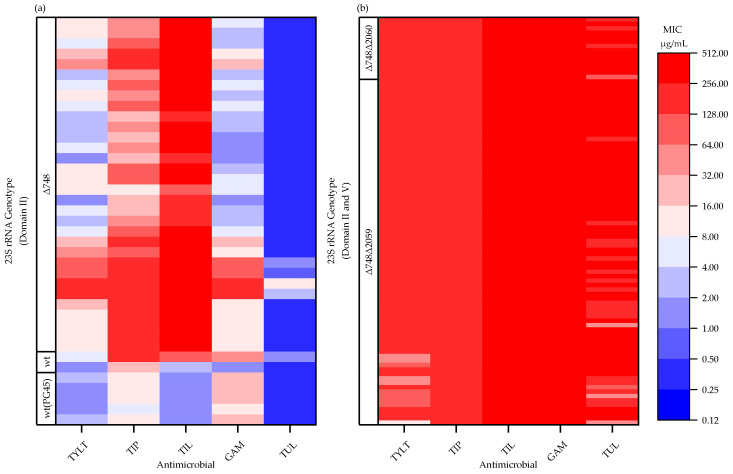
Minimum inhibitory concentrations (MIC) of *Mycoplasma bovis* isolates (*n* = 126) for tylosin (TYLT), tilmicosin (TIL), tildipirosin (TIP), gamithromycin (GAM), and tulathromycin (TUL), and the corresponding 23S rRNA genotype: (**a**) wildtype (wt) or mutations in domain II only (Δ748); (**b**) mutations in domain II and V (Δ748Δ2059, Δ748Δ2060). The MIC values for the five *M. bovis* PG45 replicates [wt(PG45)] are presented. TYLT, TIL, and TIP have a 16-membered core structure; whereas, GAM and TUL have a 15-membered core structure.

Among the 126 isolates analyzed, mutations (single nucleotide polymorphisms, SNPs) were located in hairpin 35 of domain II (nucleotide 748; *E. coli* numbering used throughout) and the peptidyl transferase loop of domain V (nucleotide 2059 and 2060) of the 23S rRNA (*rrl*) gene alleles. The 23S rRNA genotype was assigned based on alleles observed at position 748, 2059, and 2060 (Table 3). As there are up to two copies of the *rrl* gene reported for *M. bovis*, an isolate was categorized as having a change (Δ) if a mutation occurred in at least one copy of the gene. The *M. bovis* PG45 reference genome was considered as the reference (wildtype) and two isolates (1.6%) were identical to this 23S rRNA genotype. Most isolates (73.0%; 92/126) had mutations in domains II and V (Δ748Δ2059 or Δ748Δ2060); whereas, 25.4% (32/126) had a single mutation in domain II (Δ748). There were no isolates with lone mutations in domain V. All isolates with a single mutation at Δ748 were susceptible to TUL (MIC ≤ 16 µg/mL); resistant to TIP and TIL (MIC ≥ 8 µg/mL); and either susceptible (MIC ≤ 4 µg/mL) or resistant (MIC ≥ 8 µg/mL) to GAM and TYLT (Figure 1a). Isolates that had accumulated mutations in both domain II and V (Δ748Δ2059 or Δ748 Δ2060) were resistant to all five macrolides (Table 3 and Figure 1b).

No dose dependent effect was apparent within a given genotype (i.e., Δ748 only) for those with a single mutant allele (i.e., G748A) or a combined mutant/wildtype allele (i.e., G748, G748A). The MIC values for isolates with Δ748 only 23S rRNA genotype, with a single mutant allele, ranged from 1 to 128 µg/mL for GAM and TYLT, and 0.25 to 8 µg/mL for TUL. Isolates with combined alleles had consistently lower MIC values of 8, 8–16, and 0.25 µg/mL for GAM, TYLT, and TUL, respectively, which were within the MIC range for isolates with a single mutant allele. For isolates with the Δ748Δ2059 23S rRNA genotype, those with combined alleles had MIC values ranging from 8 to ≥128 µg/mL for TYLT compared to ≥128 µg/mL with a single mutant allele. Regardless of allelic composition, the MIC values for isolates with Δ748Δ2060 23S rRNA genotypes were ≥64 µg/mL.

The 23S rRNA genotypes were grouped based on the presence of mutations in domain II only versus domain II and V. The results were reported as % resistant with 95% confidence interval (95% CI) for a proportion (Table 4). The two isolates with wildtype 23S rRNA genotypes were susceptible to TUL and TYLT (0, 0–0.66), and 1 of 2 were resistant to GAM and TIL (0.5, 0.09–0.91). Isolates with mutations in domain II only (Δ748 only) had a similar proportion of isolates resistant to GAM (0.41, 0.26–0.58) and TYLT (0.59, 0.42–0.74) compared to TUL (0, 0–0.11). An additional mutation in domain V at positions 2059 (Δ748Δ2059) or 2060 (Δ748Δ2060) resulted in all isolates being resistant to all five macrolides. All isolates were resistant TIP, regardless of genotype.

The level of agreement in the classification of resistance between the presence of a mutation in domain V in the 23S rRNA genotype and phenotype (MIC values) varied by macrolide. The kappa correlation coefficient was perfect (1.000) for TUL, moderate (0.676) for GAM, weak (0.536) for TYLT, essentially nonexistent (0.042) for TIL, and could not be determined for TIP because all isolates were resistant regardless of the genotype. Despite these differences, all isolates with a mutation in domain V of the 23S rRNA genotype (Δ748Δ2059 and Δ748Δ2060) were resistant to all macrolides. However, mutations in domain V also occurred in the presence of a mutation in domain II at position 748.

### 2.4. L4 and L22 Ribosomal Proteins

All isolates had a nonsynonymous mutation Gln93His (*M. bovis* PG45 number; equivalent to Gln90His using *E. coli* numbering) in the L22 ribosomal protein. There were multiple nonsynonymous L4 mutations: Ser18Thr, Thr43Ala, Ala44Thr, Glu50Thr, Ala51Thr, Ala51Ser, Ser55Ala, Thr57Ala, Val69Ala, Ala70Thr, Glu75Ala, Ala86Thr, and Ala101Thr (*M. bovis* PG45 numbering) with three different nonsynonymous mutations at two positions in proximity to the MLS_B_ binding pocket Gly185Arg, Gly185Ala, Thr186Pro (*M. bovis* PG45 numbering; equivalent to position 64 and 65 using *E. coli* numbering, respectively).

Twelve isolates had a nonsynonymous mutation in the L4 ribosomal protein in residues proximal to the MLS_B_ binding pocket. Four had two nonsynonymous mutations Gly185Ala and Thr186Pro, and eight had a single nonsynonymous mutation Gly185Arg (Table 5). All isolates had Gln93His mutations in L22 as well. All isolates with the two nonsynonymous mutations (Gly185Ala and Thr186Pro) also had mutations in domain II of the 23S rRNA gene (Δ748). The eight isolates with a single nonsynonymous mutation (Gly185Arg) had various 23S rRNA genotypes: wildtype (*n* = 1), Δ748 (*n* = 1), and Δ748Δ2059 (*n* = 6). Overall, isolates with a Δ748 mutation in the 23S rRNA gene and mutations in L4 and L22 near the MLS_B_ binding pocket were resistant (MICs ≥16 µg/mL) to GAM, TIL, TIP and TYLT; but susceptible (MICs ≤ 8 µg/mL) to TUL.

## 3. Discussion

This study was unique in that it assessed the concordance between the genotypes and phenotypes of *M. bovis* for antimicrobial resistance (AMR) to five macrolides used to control and treat bovine respiratory disease in feedlot cattle. Of note was the inclusion of TUL, which is the most commonly used antimicrobial for BRD prophylaxis, but a pharmaceutical that has not been assessed in previous genotype–phenotype AMR studies [13,31,32,47]. This is salient because even though macrolides have a similar antibacterial mode of action, they differ in the size of the macrocyclic lactone ring and associated side-chains [48]. As a result, each macrolide has a slightly different binding affinity for domains II and V of 23S rRNA. Thus, one or more mutations within these domains may lead to very different antimicrobial susceptibility testing (AST) results. Exemplars are TUL, TIL, and TIP, where a single mutation in domain II (Δ748) conferred resistance to TIL and TIP, but not to TUL. This is consistent with the modeling of the *E. coli* ribosome, wherein TUL interacts primarily at A2058 of 23S rRNA, but is too small to span the ribosomal tunnel and interact with G748 in domain II [48]. This finding is of interest because previous genotype studies did not include TUL.

Within the 23S rRNA gene, mutations in domain V occurred at position 2059 or 2060, but not both. In contrast, Lerner et al. [31] identified two isolates with mutations in both *rrl* alleles in domain V, but at different positions (2058 and 2059). Furthermore, others have reported mutations at position 2058 in *M. bovis* [31,47,49], an outcome that was not found in the current study. Isolates with differing alleles at a given position in domain V were resistant to all five macrolides, which is consistent with a previous study in which *Mycoplasma* spp. having a heterozygous mutation in domain V conferred resistance [33]. Additionally, mutations at position 2060 have been reported in *M. bovis* isolates that are resistant to lincomycin [32], an antimicrobial with a mechanism of action similar to macrolides [7,26]. These differences in position, albeit in close proximity to one another, could reflect differences in the selective pressure of specific antimicrobials as a result of differences in use across production systems. Despite these differences, the increased resistance of *M. bovis* to macrolides as a result of mutations in domain II and domain V is consistent with previous reports [13,31,32,47].

Overall, concordance was observed between 23S rRNA genotype and AMR phenotype, which highlights the utility of molecular targets as a viable alternative to in vitro AST. Isolates with combined mutations in domain II and V binding sites of 23S rRNA gene (Δ748Δ2059 and Δ748Δ2060) all demonstrated resistance to TUL, GAM, and TYLT. Whereas regardless of genotype, >99% of all isolates were resistant to TIP and TIL. The accumulation of SNPs in domain II and V of the 23S rRNA gene and the relationship to increasing MIC values, and therefore inferred resistance, has been reported for TYLT and TIL in *M. bovis* by Hata et al. [13]. Lui and Douthwaite [50] also demonstrated that monomethylation at positions G748 and A2058 acted synergistically to increase TYLT resistance. In both the Lerner et al. [31] study and the current study, the existence of mutations in both the II and V domains correlated with MICs for TYLT and TIL that were indicative of clinical resistance. However, it has also been reported that some *M. bovis* isolates with elevated MICs to TYLT and TIL only have a mutation in domain V, without a concurrent mutation at position 748 [31,47], while others had a change at 748 without a mutation in domain V [32].

Given that TIP and TIL are both derivatives of TYLT, the similarities in the level of resistance to these macrolides is not surprising. These three macrolides vary in the groups that decorate C5, C6, and C14 of their shared 16-membered core structure. As high levels of resistance (>99%) to both TIL and TIP was present, it was difficult to correlate phenotype and genotype. However, as per previous reports [9,10,16,17] the very high MIC values for these two antimicrobials indicate that they are unsuitable for treating mycoplasmosis in cattle.

The associations of mutations in the L4 and L22 ribosomal proteins with susceptibility phenotypes were less clear than those of domains II and V of the 23S rRNA gene. Zhao et al. [51] reported that mutations in these ribosomal proteins lead to increased macrolide resistance in *M. pneumonia*. In the current study, mutations in L4 and L22 were associated with elevated MICs for GAM, TYLT, TIP, and TIL. Given that these ribosomal proteins form the narrowest constriction of the protein exit tunnel [52], with both having loops that extend adjacent to macrolide binding sites [53], the presence of mutations is consistent with the AST phenotypes. All isolates (*n* = 126) also had mutations in L22 relative to the type strain, a result more prevalent than reported by Lerner et al. [31], where the nonsynonymous mutation Gln90His (*E. coli* numbering) in L22 was observed in 75% of isolates. Again, these differences across studies are likely related to increased antimicrobial selection pressure placed on the western Canadian isolates.

There was a very low prevalence of the *M. bovis* type strain PG45 genotype (1.6%) in this study compared to Hata et al. [13], who observed this genotype in 12.3% of 203 bovine isolates from Japan. Lerner et al. [31] found that this genotype in about half of the 54 isolates from cattle originating in the Middle East, Europe, and Australia. Variation in the proportion of wildtype *M. bovis* isolates circulating within cattle populations is undoubtedly related to differences in cattle production systems and antimicrobial use. In western Canada, most beef calves are weaned in the fall of the year and sold at auctions where they are commingled with cohorts from other farms. These newly weaned calves are then transported to feedlots where they are processed on-arrival. In addition to these stressors, these events occur when the weather can be also be inclement. Therefore, calves deemed to be at high-risk of developing BRD are administered macrolides, often TUL, on-arrival. Our data indicate that over time this practice has selected against wildtype genotypes and for the emergence of macrolide resistant genotypes. Significantly, not only has macrolide resistance in western Canadian feedlot cattle been increasing, it is also not uncommon to recover macrolide resistant *M. bovis* isolates from the nasopharynx of healthy cattle at feedlot arrival [11]. While feedlots could rotate macrolides with tetracyclines or florfenicol, as a strategy to reduce resistance, this practice requires timely AST data or otherwise it may exacerbate antimicrobial resistance.

The study had a number of potential weaknesses. The wildtype 23S rRNA genotype essentially served as a control group; however, there were only two isolates in this group. This was unfortunate since one of two wildtype isolates were resistant to GAM and TIL, and both resistant to TIP. Additionally, control testing of healthy animals was not performed at the time of sampling diseased or dead animals. However, this study was not intended as a survey of macrolide susceptibility, but rather an investigation of the relationship between genotype and phenotype. Therefore, the healthy animals were sampled with the intent of culturing phenotypically susceptible isolates to serve as a basis of comparison to the abundance of resistant isolates derived from dead cattle. The other weaknesses were that the isolates were not uniformly spread over all 12 production years, and most isolates came from dead animals that had received extensive antimicrobial therapy prior to death. The study, however, also had some notable strengths. The relatively large number of isolates came from cattle that were sourced from a broad geographic area; samples were collected over 12 production years; from multiple anatomical locations; and from healthy, diseased and dead cattle.

Conventional antimicrobial susceptibility testing for *M. bovis* is time-consuming and technically demanding, making it unsuitable for monitoring antimicrobial resistance in real-time within a feedlot. This is an issue because prudent use guidelines for antimicrobial use are predicated on AST. This study, and others, have shown a clear linkage between genotypes and macrolide resistance, providing an avenue for developing a rapid, accurate, and cost-effective molecular based test for *M. bovis*, similar to what has been done for *Mycoplasma genitalium* [34,54,55]. This test could be used to assess *M. bovis* isolates obtained from nasapharyngeal swabs, or for conducting pen-level AST surveillance by testing isolates found in shared watering bowls.

## 4. Materials and Methods

### 4.1. Animals and Sample Collection

*Mycoplasma bovis* isolates were cultured from a cross-section of clinical samples derived from different anatomical regions (nasopharynx, lung, and joint) of western Canadian feedlot cattle over 12 production years (2006–2018). Deep nasopharyngeal swabs from live cattle were taken in accordance with Animal Use Protocols (#20070023; #20170021) approved by the University of Saskatchewan’s Animal Research Ethics Board and Lethbridge Research Center’s Animal Care Committee (#1641).

Sampling was performed as described in Jelinski et al. [11]. Briefly, doubled-guarded uterine swabs (Reproduction Resources, Walworth, WI, USA) were used to obtain deep nasopharyngeal (DNP) samples from healthy and diseased cattle. The diseased cattle were identified by feedlot personnel trained in recognizing the clinical signs of BRD (dyspnea, depression, nasal discharge, anorexia, and fever). Swabs were immediately placed in Ames media (Mai, Ames Media, Product 49203, Spring Valley, WI, USA).

All other swabs or tissues were collected from animals purposively sampled by feedlot veterinarians recruited to provide clinical case material for the study. Samples were obtained by the veterinarians from animals that on postmortem examination were found to have pathological lesions consistent with *M. bovis* pneumonia or chronic pneumonia and polyarthritis syndrome (CPPS). Specifically, the lungs had gross pathology consistent with caseonecrotic pneumonia and/or chronic bronchopneumonia. A minimum 3 × 3 cm lung sample was excised and if septic arthritis was concurrently observed, then the diseased joints were sampled by swabbing, aspirating synovial fluid, or excising synovial tissue.

Tissue and fluid specimens were stored at –20 °C, and batch shipped by courier. Upon receipt, samples were stored at –80 °C until culturing. For each sample, the following metadata were recorded: date of sampling, type of sample (swab, tissue, joint fluid), anatomical location (nasopharynx, lung, joint), and disease status (healthy, diseased, dead).

### 4.2. Mycoplasma Culture and Isolation

Selective culture was performed on the DNP swabs and on swabs of the cut tissue surfaces as previously described by Jelinski et al. [11]. Due to the extended time span of sample collection, there were slight differences in isolation methods and media over the course of the study. Samples collected between 2006 to 2008 were cultured using Hayflick’s media (made in-house), whereas in subsequent years samples were cultured using pleuropneumonia-like organism (PPLO) broth and agar (BD Difco, Fisher Scientific, Waltham, MA, USA), supplemented with 10 g/L yeast extract (BD Diagnostic Systems, Fisher Scientific, Waltham, MA, USA), and 20% horse serum (Invitrogen, Fisher Scientific) [11,56]. Where specified, the media was supplemented with 0.05% thallium (I) acetate, 500 U/mL penicillin G, and/or 0.5% sodium pyruvate (Sigma-Aldrich, St. Louis, MO, USA).

Cultures derived from samples were filtered through 0.45 and 0.20 µm filters (Basix, VWR International, Radnor, PA, USA), and were used to inoculate PPLO broth with 0.05% thallium (I) acetate, 500 U/mL penicillin G, and 0.5% sodium pyruvate, and grown in a 5% CO_2_ atmosphere with 75% humidity at 37 °C. Cultures with visible growth were streaked onto PPLO agar with 0.05% thallium (I) acetate and 500 U/mL penicillin G and incubated for 3–6 days. An isolated colony with characteristic “fried-egg” morphology was picked, replated on PPLO agar, and incubated for 72 h. Up to three individual colonies per sample were used to inoculate separate aliquots of PPLO broth with 0.05% thallium (I) acetate and 500 U/mL penicillin G. After 48 h of growth, each culture was separately stored in PPLO with glycerol (20%, *v*/*v*) at –80 °C. From the three possible cultures, a single culture was chosen to inoculate PPLO media for DNA extraction and antimicrobial susceptibility testing.

### 4.3. DNA Extraction and Identification

*Mycoplasma bovis* isolates were grown in PPLO media for 48 h and the genomic DNA was extracted using the GenElute Bacterial Genomic DNA Kit (Sigma-Aldrich, St. Louis, MO, USA). The DNA was extracted following manufacturer’s instructions for Gram negative bacteria with the final elution buffer replaced with 10 mM Tris (pH 8.5). Extracted genomic DNA was assessed for quality using gel electrophoresis and quantified fluorometrically using Qubit (Thermo Fisher Scientific, Waltham, MA, USA). Cultures were confirmed as *M. bovis*, based on confirmation of the presence of *uvrC* [57] and sequencing of the 16S rRNA gene [58]. The 16S rRNA amplicon was purified using a QIAquick PCR purification kit (Qiagen, Nevlo, Netherlands) and sent to Macrogen (Seoul, South Korea) for Sanger sequencing with the amplification primers. Forward and reverse sequences were assembled and edited using the Staden Package (version 1.6-r, http://staden.sourceforge.net/). The resulting sequences were compared to the National Center for Biotechnology Information (NCBI) nonredundant nucleotide database (nr) using BLASTn.

### 4.4. Whole Genome Sequencing and Assembly

Genomic DNA was prepared for sequencing using Illumina Nextera XT DNA Library Preparation (Illumina Inc., San Diego, CA, USA) and sequenced on a Illumina MiSeq platform using the MiSeq v2 Reagent Kit to generate 250 bp paired-end reads. Illumina reads were trimmed using Trimmomatic v0.38 [59] with settings slidingwindow:5:15 leading:5 trailing:5 and minlen:50. Genomes were assembled with *M. bovis* PG45 as the reference genome (CP002188) using BWA-MEM v0.7.10-r789 [60] with default settings, producing SAM formatted assemblies. SAMtools [61] was used to convert the assemblies to BAM files and then sort and index for further processing. The Picard v2.18.4-SNAPSHOT [62] marked and removed duplicate reads from the BAM file. The Genome Analysis ToolKit v3.4-46-gbc02625 was used to perform local indel realignment and base quality score recalibration to improve the alignment quality, according to GATK best practices pipeline [63]. Consensus sequences for each assembly were created from each BAM file using bcftools [61]. This was performed by piping results from bcftools mpileup to bcftools call to create a vcf file for each BAM file. Each vcf file was used as input for vcfutils vcf2fq to generate a consensus fastq file. The fastq files were converted to fasta files using a bash script.

Genes encoding for 23S rRNA (*rrl3* and *rrl4*), L4 (*rplD*), and L22 (*rplV*) ribosomal proteins were identified using BLASTn [64] to compare *M. bovis* strain PG45 genes to the assembled genomes. For *rrl3* and *rrl4*, the closest matching sequence to the start of the genome being analyzed was identified as *rrl3*, the furthest as *rrl4*. As two start sites have been proposed for ribosomal protein L4, for the purposes of this study the position of *rplD* and overall numbering was based on locus ID MBOVPG45_0263. Extraction of gene sequences was performed using the start and ends of the match as reported by BLASTn for input to SAMtools faidx [61]. Genes of interest extracted from each isolate were aligned with the equivalent region in the *M. bovis* PG45 reference genome (CP002188.1) in Geneious Prime 2020.0.5 (https://www.geneious.com) using MUSCLE to identify SNPs with a minimum variant frequency of 0.01. For L4 and L22 ribosomal protein gene alignments, they were translated using the *Mycoplasma* spp. genetic code. To verify the nucleotide composition in *rrl3* and *rrl4* at positions within hairpin 35 in domain II and the peptidyl transferase loop in domain V within the MLS_B_ binding pocket [65], the SAM files were queried for ambiguity to determine the representative nucleotide(s). In cases of ambiguity, the percent of reads for a given allele was >20%. The raw paired reads for the isolates used in this study are available at NCBI SRA (www.ncbi.nlm.nih.gov/sra) with BioProject ID PRJNA642970.

The *M. bovis* sequences were aligned to their respective 23S rRNA (*rrlA*), L4 (*rplD*), or L22 ribosomal protein (*rplV*) genes isolated from the *E. coli* K12 substrain MG1655 genome to determine equivalent positioning to generate numbering to allow for comparison between different studies and bacterial species.

### 4.5. Antimicrobial Susceptibility Testing

Antimicrobial susceptibility (AST) was determined using a microdilution assay, in a Sensititre™ (Trek Diagnostics, Oakwood, GA, USA) plate format and a customized panel designed to assess the antimicrobials most commonly used in North American feedlots for the treatment and control of BRD. The panel consisted of ten antimicrobials as described by Jelinski et al. [11], five of which were macrolides: tildipirosin (TIP; 0.12–128 µg/mL), gamithromycin (GAM; 0.25–256 µg/mL), tulathromycin (TUL; 0.25–256 µg/mL), tilmicosin (TIL; 1–256 µg/mL), and tylosin tartrate (TYLT; 1–128 µg/mL). AlamarBlue (ThermoFisher Scientific, DAL1100), a color redox indicator, was used to assess growth in each well based on a blue to pink color transition.

The AST procedure began by inoculating an *M. bovis* isolate previously stored at –80 °C in 20% glycerol into PPLO broth with 0.5% pyruvate and incubating for 72 h at 5% CO_2_ with 75% humidity at 37 °C. Broth cultures were then subcultured into neat PPLO (without antibiotics) and incubated for 24 h. Following incubation, the optical density (OD) at 450 nm was determined using a NanoDrop One Spectrophotometer (Fisher Scientific, Waltham, MA, USA) and the culture adjusted to an OD_450_ = 0.1. The adjusted culture was diluted up to 100×, and 120 µL of the diluted culture used to inoculate 6 mL of PPLO in 2× alamarBlue. Each well of a Sensititre™ plate was inoculated to a final concentration of 10^3^ to 5 × 10^5^ CFU/mL with 50 µL of culture in 2× alamarBlue in 50 µL of media with each of antimicrobials within a series of Sensititre wells (final working concentration of alamarBlue: 1×; final well volume: 100 µL). Plates were sealed with a CO_2_ permeable film, and incubated for 48–72 h. Minimum inhibitory concentrations (MICs) were determined by visual assessment of plates at 48 and 72 h, based on a blue to pink colour change. The *M. bovis* reference strain (*Mycoplasma bovis* ATCC^®^ 25523™) was tested five times for quality control.

### 4.6. Clinical Breakpoints

As there are no established macrolide breakpoints for *M. bovis,* they were extrapolated from other members of the bacterial BRD complex (i.e., *Mannheimia haemolytica*, *Pasteurella multocida*, *Histophilus somni*) and human *Mycoplasma* spp., as described previously in Jelinski et al. [11]. The resistance breakpoints were ≥8 µg/mL for TIP, GAM, TIL, and TYLT, and ≥32 µg/mL for TUL.

### 4.7. Statistical Analysis

As *rrl3* and *rrl4* genes in the reference sequence for *M. bovis* PG45 differ by only a single nucleotide, alleles in each isolate could not be assigned to a specific locus. Instead, allele(s) at a given position were reported and isolates were grouped into genotypes according to the presence of mutation(s) in domain II and V. This created four 23S rRNA genotype groups: wildtype, Δ748 only, Δ748Δ2059, and Δ748Δ2060.

As phenotypically resistant and susceptible isolates were derived from cattle in each health status cohort (healthy, diseased, and dead), all isolates were analyzed together regardless of their source. Confidence intervals were calculated using the Wilson score interval method for estimating intervals for proportions. The confidence intervals were used to represent the antimicrobial resistance for a given 23S rRNA genotype using Epitools [66]. To assess the agreement in classification of resistance between the presence of a mutation in domain V of the 23S rRNA genotype and phenotype (MIC value), the Cohen’s kappa statistic interpretation criteria (value, level of agreement): 0–0.20, none; 0.21–0.39, minimal; 0.40–0.59, weak; 0.60–0.79, moderate; 0.80–0.90, strong; >0.90, almost perfect [67] were calculated using a commercial statistical program (SPSS 26, IBM SPSS Statistics version 26, IBM Corporation, Armonk, NY, USA). All descriptive statistics were calculated using a commercial spreadsheet software (Microsoft Excel version 15; Microsoft Corporation, Redmond, Washington, WA, USA).

## 5. Conclusions

Given that antimicrobials are the primary preventative and therapeutic tool to combat *M. bovis* infections in feedlot cattle, ongoing assessment of antimicrobial susceptibility remains crucial to maintaining the utility of these drugs and facilitating antimicrobial stewardship practices. However, the comparatively slow growth of *M. bovis* yields longer turn-around times when exclusively using culture-based methods of assessment, which can impede timely decision making on antimicrobial use. In our study, we were able to identify mutations in domains II and V of the 23S rRNA genes that are associated with increased resistance to macrolides which show a clear linkage between genotype and phenotypic macrolide resistance (AST). These findings add strong support for utilizing rapid, accurate, and cost-effective molecular based tests for assessing the susceptibility of *M. bovis* to macrolides.

## Figures and Tables

**Table 1:** *Mycoplasma bovis* isolates (*n =* 126) by anatomical location, health status, and production year.

	Production Year								
	2006	2007	2008	2014	2015	2016	2017	2018	Total
Anatomical Location									
Joint		1	1	1	1	14	11	6	35
Lung				3	3	17	15	6	44
Nasopharynx	5	28					9	5	47
Total	5	29	1	4	4	31	35	17	126
Health Status									
Healthy	2	14					9	5	30
Diseased	3	12							15
Dead		3	1	4	4	31	26	12	81
Total	5	29	1	4	4	31	35	17	126

**Table 2:** Number of *Mycoplasma bovis* isolates (*n* = 126) with a resistant (R) or susceptible (S) phenotype by health status.

Health Status	Phenotype (% Resistant)						
		GAM	TIL	TIP	TUL	TYLT	Total
Healthy	R/S (%R)	19/11 (63.3)	30/0 (100)	30/0 (100)	11/19 (36.7)	22/8 (73.3)	30
Diseased	R/S (%R)	9/6 (60.0)	15/0 (100)	15/0 (100)	9/6 (60.0)	11/4 (73.3)	15
Dead	R/S (%R)	78/3 (96.3)	80/1 (98.8)	81/0 (100)	72/9 (88.9)	78/3 (96.3)	81
Total	R/S (%R)	106/20 (84.1)	125/1 (99.2)	126/0 (100)	92/34 (73.0)	111/15 (88.1)	126

GAM—gamithromycin, TIL—tilmicosin, TIP—tildipirosin, TUL—tulathromycin, and TYLT—tylosin.

**Table 3:** Comparison of 23S rRNA genotypes and the number (%) of *Mycoplasma bovis* isolates (*n* = 126) resistant to the five macrolides tested.

Genotype	23S rRNA Gene Alleles^+^			Percent (*n*) of Isolates	Phenotype^#^ (% Resistant)				
	Domain II	Domain V			GAM	TIL	TIP	TUL	TYLT
	748	2059	2060						
Wildtype*	G748	A2059	A2060	1.6 (2)	1 (50)	1 (50)	2 (100)	0 (0)	0 (0)
Total				1.6 (2)	1 (50)	1 (50)	2 (100)	0 (0)	0 (0)
Δ748 only	G748, G748A^‡^			4.0 (5)	5 (100)	5 (100)	5 (100)	0 (0)	5 (100)
	G748A			21.4 (27)	8 (29.6)	27 (100)	27 (100)	0 (0)	14 (51.9)
Total				25.4 (32)	13 (40.6)	32 (100)	32 (100)	0 (0)	19 (59.4)
Δ748Δ2059	G748, G748A^‡^	A2059, A2059G^‡^		0.8 (1)	1 (100)	1 (100)	1 (100)	1 (100)	1 (100)
	G748A	A2059, A2059G^‡^		2.4 (3)	3 (100)	3 (100)	3 (100)	3 (100)	3 (100)
	G748A	A2059, A2059C^‡^		2.4 (3)	3 (100)	3 (100)	3 (100)	3 (100)	3 (100)
	G748A	A2059, A2059T^‡^		7.1 (9)	9 (100)	9 (100)	9 (100)	9 (100)	9 (100)
	G748A	A2059G		49.2 (62)	62 (100)	62 (100)	62 (100)	62 (100)	62 (100)
Total				61.9 (78)	78 (100)	78 (100)	78 (100)	78 (100)	78 (100)
Δ748Δ2060	G748A		A2060, A2060C^‡^	0.8 (1)	1 (100)	1 (100)	1 (100)	1 (100)	1 (100)
	G748A		A2060C	7.9 (10)	10 (100)	10 (100)	10 (100)	10 (100)	10 (100)
	G748A		A2060G	2.4 (3)	3 (100)	3 (100)	3 (100)	3 (100)	3 (100)
Total				11.1 (14)	14 (100)	14 (100)	14 (100)	14 (100)	14 (100)

* *Mycoplasma bovis* PG45 is designated as wildtype genotype. ^#^ GAM—gamithromycin, TIL—tilmicosin, TIP—tildipirosin, TUL—tulathromycin, and TYLT—tylosin. ^‡^ Representative of a combined wildtype and mutant allele. ^+^ Positioning of the alleles is based on *Escherichia coli* numbering.

**Table 4:** Number and proportion of M*ycoplasma bovis* isolates (*n* = 126) resistant (R) or susceptible (S) by 23S rRNA genotype. The 95% binomial proportion confidence interval (Wilson score) is an interval estimate of the probability of the isolate being resistant if it has a particular 23S rRNA genotype.

		23S rRNA Genotype^+^			
		Wildtype	Δ748 only	Δ748Δ2059	Δ748Δ2060
TUL	R/S	0/2	0/32	78/0	14/0
Proportion (95% CI)		0 (0–0.66)	0 (0–0.11)	1 (0.95–1)	1 (0.78–1)
GAM	R/S	1/1	13/19	78/0	14/0
Proportion (95% CI)		0.50 (0.09–0.91)	0.41 (0.26–0.58)	1 (0.95–1)	1 (0.78–1)
TYLT	R/S	0/2	19/13	78/0	14/0
Proportion (95% CI)		0 (0–0.66)	0.59 (0.42–0.74)	1 (0.95–1)	1 (0.78–1)
TIL	R/S	1/1	32/0	78/0	14/0
Proportion (95% CI)		0.50 (0.09–0.91)	1 (0.89–1)	1 (0.95–1)	1 (0.78–1)
TIP	R/S	2/0	32/0	78/0	14/0
Proportion (95% CI)		1 (0.34–1)	1 (0.89–1)	1 (0.95–1)	1 (0.78–1)
Total		2	32	78	14

GAM—gamithromycin, TIL—tilmicosin, TIP—tildipirosin, TUL—tulathromycin, and TYLT—tylosin. ^+^ Positioning of the alleles is based on *Escherichia coli* numbering.

**Table 5:** Presence of ribosomal protein mutations in different 23S genotype groups and corresponding minimum inhibitory concentrations (MIC) values.

23S rRNA Genotype^+^	MIC (µg/mL)					Ribosomal Proteins^‡^	
	GAM	TIL	TIP	TUL	TYLT	L4	L22
wildtype (PG45)	8–16	1	4–8	0.25	1–2	Gly185, Thr186	Gln93
wildtype	32	64	>128	1	4	Gly185Arg	Gln93His
Δ748	128	>256	>128	2	128	Gly185Ala, Thr186Pro	Gln93His
	128	>256	>128	8	128	Gly185Ala, Thr186Pro	Gln93His
	64	>256	>128	0.5	64	Gly185Ala, Thr186Pro	Gln93His
	64	>256	>128	1	64	Gly185Ala, Thr186Pro	Gln93His
	16	256	128	0.25	32	Gly185Arg	Gln93His
Δ748Δ2059	>256	>256	>128	>256	>128	Gly185Arg	Gln93His
	>256	>256	>128	256	>128	Gly185Arg	Gln93His
	>256	>256	>128	>256	>128	Gly185Arg	Gln93His
	>256	>256	>128	128	64	Gly185Arg	Gln93His
	>256	>256	>128	128	64	Gly185Arg	Gln93His
	>256	>256	>128	32	64	Gly185Arg	Gln93His

^+^ Positioning of the alleles is based on *Escherichia coli* numbering. **^‡^** Positioning of amino acids is based on *Mycoplasma bovis* PG45 numbering.
